# Biomarker–questionnaire discordance in secondhand smoke exposure among Korean children: A secondary analysis of the Korean National Environmental Health Survey, 2016-2017 and 2019-2020

**DOI:** 10.18332/tid/226880

**Published:** 2026-09-04

**Authors:** Hee-jeong Lee, Sung-il Cho

**Affiliations:** 1 Department of Public Health Science, Graduate School of Public Health, Seoul National University, Seoul, Republic of Korea; 2 Institute of Health and Environment, Graduate School of Public Health, Seoul National University, Seoul, Republic of Korea

**Keywords:** secondhand smoke, urinary cotinine, discordance, cross-sectional study, children

## Abstract

**INTRODUCTION:**

Questionnaire-based assessment of secondhand smoke (SHS) exposure may not fully capture children's internal nicotine burden. We assessed concordance between parent-reported SHS exposure and urinary cotinine classifications and examined correlates of discordance by developmental stage and survey cycle.

**METHODS:**

This secondary analysis included children aged 3–11years with urine specimens and parent-completed questionnaire data from two independent cross-sectional samples in the Korean National Environmental Health Survey (KoNEHS 2016–2017 and 2019–2020; N=2619). Pooled analyses were unweighted; cycle-specific weighting was examined in sensitivity analyses. Concordance between parent-reported SHS exposure (≥1 time/week) and urinary cotinine-based classifications at three prespecified thresholds [at or above the limit of detection (LOD), ≥3 ng/mL, and ≥5 ng/mL] was evaluated using sensitivity, specificity, Cohen's κ, and McNemar's test. Correlates of questionnaire-negative/biomarker-positive discordance were examined using multivariable logistic regression.

**RESULTS:**

Concordance was negligible across thresholds and age groups (κ range: -0.02–0.01; McNemar p<0.001 for most strata). Sensitivity ranged from 7.1% to 16.1%, and at least 83.9% of biomarker-positive children were questionnaire-negative. Parent-reported SHS exposure was lower in Cycle 4 among children aged 3–5 and 6–8 years, whereas urinary cotinine concentrations were higher across all age groups. Among children aged 3–5 and 6–8 years without parent-reported SHS exposure, cotinine concentrations were higher in those with at least one current parental smoker than in those with no current parental smoker. At ≥LOD, questionnaire-negative/biomarker-positive discordance was associated with younger age (3–5 vs 9–11 years: adjusted odds ratio, AOR=1.64; 95% CI: 1.20–2.24) and with Cycle 4 (vs Cycle 3: AOR=2.09; 95% CI: 1.59–2.75).

**CONCLUSIONS:**

Parent-reported SHS exposure and urinary cotinine classifications showed marked discordance. These measures captured related but non-interchangeable information; urinary cotinine may complement questionnaires when characterizing children's internal nicotine burden.

## INTRODUCTION

Children are more susceptible to secondhand smoke (SHS) exposure than adults^1^ because of higher ventilation rates relative to body weight, immature detoxification systems, and greater time spent in indoor environments. SHS exposure in childhood is associated with respiratory morbidity, impaired lung development, and allergic conditions^2,3^. Household smoking is widely recognized as a major source of SHS exposure in domestic settings, particularly for children spending time at home^4,5^. Reliable assessment of SHS exposure in children is therefore important for tobacco-control surveillance and for evaluating smoke-free household interventions.

Exposure assessment in population surveys commonly relies on parent-completed questionnaires. However, questionnaire-based measures may not fully capture children’s internal nicotine burden because of recall bias, social desirability bias, or limited awareness of indirect exposure pathways^6-8^. Urinary cotinine, a biomarker of recent internal nicotine burden, is also widely used in biomonitoring studies^9,10^. Discordance between parent-reported SHS exposure and urinary cotinine-based classifications has been documented across diverse settings^6,11,12^, but its magnitude, directionality, and correlates across developmental stages remain poorly characterized. Prior studies suggest that discordance may reflect reporting bias, construct divergence between questionnaires and biomarkers, and changes in exposure environments^13-15^. These mechanisms have different implications for surveillance design.

South Korea presents a particularly relevant context. Although overall adult smoking prevalence has declined, male smoking rates remain high^16,17^, and heated tobacco products (HTPs) were introduced nationally in 2017 with rapid market uptake^18,19^. The COVID-19 pandemic in 2020 also coincided with changes in children’s time-activity patterns, particularly among preschool-aged children^20^. These contextual shifts may have contributed to differences in children’s nicotine-exposure environments and in parent-reported behavior between the two survey cycles. Because the two cycles comprise independent cross-sectional samples, between-cycle differences cannot be interpreted as longitudinal trends. Nevertheless, comparisons under a harmonized analytic framework can clarify whether concordance patterns differ across survey periods.

The Korean National Environmental Health Survey (KoNEHS) incorporates standardized urinary cotinine measurements^10^ alongside detailed questionnaire data^9^. This design allows evaluation of biomarker-based and parent-reported measures within the same population-based biomonitoring framework. The present study does not aim to distinguish among the mechanisms underlying discordance, but rather to quantify its extent and structure under real-world surveillance conditions.

Using data from children aged 3–11 years in KoNEHS Cycles 3 (2016–2017) and 4 (2019–2020), we aimed to: 1) assess concordance between parent-reported SHS exposure and urinary cotinine-based classifications at multiple prespecified thresholds; 2) identify correlates of questionnaire-negative/biomarker-positive discordance; and 3) characterize age-specific associations between parental smoking and urinary cotinine concentrations.

## METHODS

### Study design and population

This secondary analysis used data from two independent cross-sectional samples from the Korean National Environmental Health Survey (KoNEHS), Cycle 3 (2016–2017) and Cycle 4 (2019–2020). KoNEHS is a nationwide human biomonitoring program in Korea, administered by the National Institute of Environmental Research (NIER). It employs a stratified, multistage probability sampling design based on the Population and Housing Census, with stratification by region and housing type^9^.

Eligible participants were children aged 3–11 years with available urine specimens and parent-completed questionnaires. Cycles 3 and 4 were selected because they were the first two KoNEHS cycles to include pediatric urinary cotinine data. Within Cycle 4, children aged 6–8 and 9–11 years were surveyed in 2019, whereas those aged 3–5 years were surveyed in 2020 during the COVID-19 pandemic. This age-specific survey timing should be considered when interpreting between-cycle differences in time-activity patterns and cotinine concentrations (Supplementary file Table S3).

Survey protocols were approved by the NIER Institutional Review Board (NIER-2016-BR-003-01; NIER-2016-BR-003-03; NIER-2018-BR-003-02), and written informed consent was obtained from parents or legal guardians. This secondary analysis of de-identified data was deemed exempt from review by the Seoul National University Institutional Review Board (IRB No. E2509/003-004).

Children were excluded if their housing type could not be consistently classified across survey cycles, or if required data on building age, socioeconomic characteristics, paternal smoking, or home ventilation were missing. Before model specification, an indicator identifying children who lived with a non-parental household smoker but had no current parental smoker was evaluated for an independent association with urinary cotinine, and multicollinearity among the candidate covariates was assessed using variance inflation factors (VIFs).

Analyses used complete cases. Because sampling weights were cycle-specific and derived from different sampling designs, pooled analyses were unweighted. Cycle-specific weighted sensitivity analyses were conducted (Supplementary file Table S5). Accordingly, pooled estimates should be interpreted as associations within the analytic sample rather than as nationally representative estimates.

### Urinary cotinine and creatinine

Urine specimens were collected using standardized protocols and analyzed at a central laboratory. Urinary cotinine was quantified by Gas Chromatography-Mass Spectrometry (GC-MS). The limit of detection (LOD) was 0.3 ng/mL in Cycle 3 and 0.2 ng/mL in Cycle 4; concentrations below the LOD were imputed as LOD/√2. Quality control procedures included pooled controls, duplicate analyses, and participation in the German External Quality Assessment Scheme (G-EQUAS).

Cotinine concentrations were natural log-transformed for regression analyses. Urinary creatinine was separately log-transformed and included as a continuous covariate rather than used to calculate a cotinine-to-creatinine ratio, to minimize potential bias associated with ratio normalization^21^. For descriptive comparisons, model-based estimates were back-transformed and reported as creatinine-adjusted geometric means (GMs) with 95% confidence intervals (CIs).

### Exposure and covariates

Parent-completed questionnaires assessed parental smoking, sociodemographic and housing characteristics, home ventilation, children’s time-activity patterns, and current allergic disease (physician diagnosis plus current symptoms or treatment). Parents reported how often the child was exposed to smoke from others’ cigarettes in indoor or enclosed spaces (none, 1–2, 3–4, or 5–6 times/week, or daily); ≥1 time/week was classified as exposure. The item did not distinguish specific settings and assessed frequency using weekly categories rather than a specified recall window. Paternal smoking was categorized as non-smoker (never/former), moderate smoker (<20 cigarettes/day), or heavy smoker (≥20 cigarettes/day); maternal smoking referred to current smoking at the time of the survey, was modeled as current versus non-current owing to its low prevalence (<1%), and did not refer to smoking during pregnancy. Household smoking status was defined as ≥1 current parental smoker.

Time-activity variables (indoor time at home, at school, and elsewhere, assessed separately for weekdays and weekends) were categorized using the cutoffs in Supplementary file Table S3 and included in cycle-specific models (Supplementary file Table S5) and in additional age-stratified sensitivity analyses. One participant with missing time-activity data was excluded from these analyses only. These variables were omitted from primary models for parsimony and from concordance models because they reflect exposure opportunity rather than reporting behavior.

Additional covariates included vigorous physical activity frequency (0–2, 3–6 times/week, or daily), housing type, building age, household income, education and occupation of both parents, and home ventilation frequency. Covariates were selected *a priori* based on biological plausibility and prior literature^7,22,23^.

### Statistical analysis

Participant characteristics were compared across age groups using Pearson’s chi-squared tests for categorical variables. Creatinine-adjusted geometric mean urinary cotinine concentrations and 95% confidence intervals were estimated from generalized linear models of log-transformed cotinine, with log-transformed urinary creatinine included as a covariate. Multivariable linear regression models examined paternal smoking status as the primary exposure and were adjusted for maternal smoking, survey cycle, sex, housing type, building age, household income, education and occupation of both parents, home ventilation frequency, vigorous physical activity frequency, current allergic disease status, and log-transformed urinary creatinine. Parent-reported SHS exposure frequency was excluded from these multivariable models to avoid conceptual overlap and potential collinearity with parental smoking variables; the cross-classification of household smoking status and parent-reported SHS exposure was evaluated in separate age-stratified multivariable models.

Age-stratified models were estimated for each developmental group (Supplementary file Table S2), and regression coefficients were reported on the log-cotinine scale; exponentiated coefficients can be interpreted as geometric mean ratios (GMRs). Differences among age-stratified paternal-smoking estimates were not interpreted as evidence of effect modification because formal interaction testing of paternal smoking by age group was not the primary analytical objective. Analyses stratified by household smoking status were also conducted, with paternal and maternal smoking variables omitted because household smoking status was derived from current paternal and maternal smoking (Supplementary file Table S1). Collinearity was assessed using VIFs, with values <2.5 considered acceptable.

Exploratory interaction models (survey cycle × age group; survey cycle × paternal smoking intensity) were fitted to examine potential heterogeneity across cycles (Supplementary file Table S4). Given multiple comparisons and the absence of prespecified directional hypotheses, interaction analyses were considered exploratory; findings were interpreted descriptively, and individual p-values were treated as nominal.

Concordance between parent-reported SHS exposure (≥1 time/week) and urinary cotinine-based classifications was evaluated at three urinary cotinine thresholds (≥LOD, ≥3 ng/mL, and ≥5 ng/mL)^24^. For descriptive purposes, parent-reported SHS exposure was treated as the index classification and each prespecified urinary cotinine threshold as an operational comparison criterion, rather than as a gold-standard definition of SHS exposure. Sensitivity, specificity, positive predictive value (PPV), negative predictive value (NPV), and Cohen’s κ were calculated to describe concordance, consistent with prior studies^25^.

Cohen’s κ quantifies agreement beyond chance but does not indicate the direction of disagreement; therefore, McNemar’s test was used to evaluate whether discordant pairs were symmetrically distributed. A significant result indicates directional asymmetry in discordance, meaning that one discordant pattern occurs more frequently than the other. Questionnaire-negative/biomarker-positive discordance was defined as the absence of parent-reported SHS exposure despite urinary cotinine at or above the prespecified threshold. These thresholds were selected to reflect a range from urinary cotinine at or above the LOD to commonly applied epidemiological cutoffs for SHS exposure in children^24^.

Correlates of questionnaire-negative/biomarker-positive discordance were examined using multivariable logistic regression restricted to biomarker-positive children (≥LOD, n=2282; ≥3 ng/mL, n=633), adjusting for age group, survey cycle, sex, education of both parents, and paternal and maternal smoking status. This restriction made the outcome conditional on meeting the specified biomarker threshold. The ≥5 ng/mL threshold was excluded from logistic analyses due to sparse cells and the resulting imprecision in the estimates. All analyses were conducted using SAS version 9.4 (SAS Institute Inc., Cary, NC, USA)^26^ and a two-sided p<0.05 was considered statistically significant.

## RESULTS

### Characteristics of the study population

**Figure 1:**
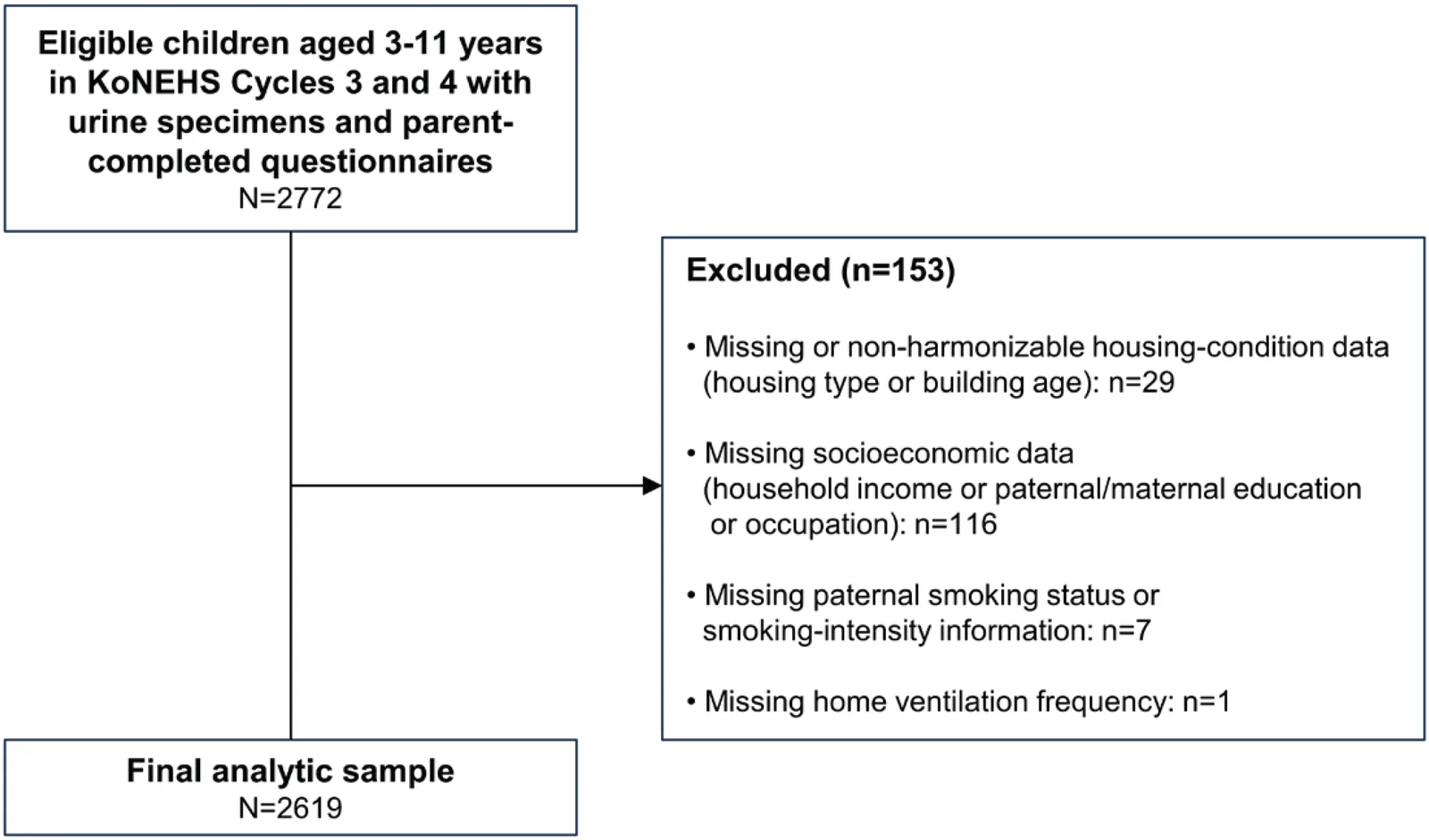
Flow chart of study sample selection

Of 2772 eligible children with urine specimens and parent-completed questionnaires, 153 with missing or non-harmonizable housing-condition, socioeconomic, paternal smoking, or home ventilation data were excluded, yielding a final analytic sample of 2619 children (Figure 1). The final sample comprised 1104 children aged 3–5 years, 754 aged 6–8 years, and 761 aged 9–11 years (Table 1). Analyses involving time-activity variables included 2618 participants because one child lacked these data. Among the covariates retained in the primary model, all variance inflation factors were below 2.5 (maximum VIF = 1.46), suggesting no substantial multicollinearity. In a separate preliminary model, the indicator of a non-parental household smoker among children with no current parental smoker was not independently associated with urinary cotinine (β= -0.009, p=0.937) and had a VIF of 1.03. Based on this evaluation and the prespecified focus on parental smoking, non-parental household smoking was not included in the primary models.

**Table 1:** Characteristics of children aged 3–11 years in a cross-sectional secondary analysis of KoNEHS Cycles 3 and 4, South Korea, 2016–2017 and 2019–2020 (N=2619)

*Variables*	*Categories*	*3–5 years (N=1104)* *n (%)*	*6–8 years (N=754)* *n (%)*	*9–11 years (N=761)* *n (%)*	*p*
**Survey cycle**	Cycle 3	550 (49.8)	414 (54.9)	409 (53.7)	0.067
	Cycle 4	554 (50.2)	340 (45.1)	352 (46.3)	
**Sex**	Male	552 (50.0)	381 (50.5)	371 (48.8)	0.774
**Housing type**	Detached	126 (11.4)	105 (13.9)	123 (16.2)	<0.001
	Apartment	888 (80.4)	554 (73.5)	559 (73.5)	
	Attached	90 (8.2)	95 (12.6)	79 (10.4)	
**Building age**	≥30 years	71 (6.4)	47 (6.2)	57 (7.5)	0.562
**Monthly household income** (10000 KRW)	<300	192 (17.4)	114 (15.1)	143 (18.8)	0.317
	300–700	732 (66.3)	519 (68.8)	509 (66.9)	
	>700	180 (16.3)	121 (16.0)	109 (14.3)	
**Paternal education level**	College or higher	903 (81.8)	580 (76.9)	546 (71.7)	<0.001
**Maternal education level**	College or higher	894 (81.0)	579 (76.8)	526 (69.1)	<0.001
**Paternal occupation**	Non-manual	551 (49.9)	363 (48.1)	337 (44.3)	0.216
	Service	178 (16.1)	134 (17.8)	145 (19.1)	
	Manual	370 (33.5)	254 (33.7)	272 (35.7)	
	Unemployed	5 (0.5)	3 (0.4)	7 (0.9)	
**Maternal occupation**	Non-manual	455 (41.2)	295 (39.1)	292 (38.4)	0.001
	Service	99 (9.0)	77 (10.2)	120 (15.8)	
	Manual	48 (4.3)	33 (4.4)	37 (4.9)	
	Unemployed	502 (45.5)	349 (46.3)	312 (41.0)	
**Paternal smoking**	Non-smoker	640 (58.0)	422 (56.0)	429 (56.4)	0.800
	Moderate smoker	340 (30.8)	241 (32.0)	234 (30.7)	
	Heavy smoker	124 (11.2)	91 (12.1)	98 (12.9)	
**Maternal smoking**	Non-smoker	1095 (99.2)	752 (99.7)	747 (98.2)	0.006
	Smoker	9 (0.8)	2 (0.3)	14 (1.8)	
**Household smoking status**	No current parental smoker	640 (58.0)	421 (55.8)	428 (56.2)	0.608
	At least one current parental smoker	464 (42.0)	333 (44.2)	333 (43.8)	
**Vigorous physical activity** (times/week)	0–2	504 (45.7)	263 (34.9)	354 (46.5)	<0.001
	3–6	485 (43.9)	405 (53.7)	336 (44.2)	
	7	115 (10.4)	86 (11.4)	71 (9.3)	
**Current allergic disease**	Any	299 (27.1)	271 (35.9)	294 (38.6)	<0.001
**Home ventilation frequency** (times/week)	1–3	727 (65.9)	533 (70.7)	552 (72.5)	0.006
	4–6	196 (17.8)	128 (17.0)	125 (16.4)	
	7	181 (16.4)	93 (12.3)	84 (11.0)	
**Parent-reported SHS exposure** (times/week)	≥1	105 (9.5)	82 (10.9)	119 (15.6)	<0.001
**Urinary cotinine** (ng/mL)	≥LOD	969 (87.8)	640 (84.9)	673 (88.4)	0.084
	≥3	245 (22.2)	183 (24.3)	205 (26.9)	0.063
	≥5	70 (6.3)	61 (8.1)	68 (8.9)	0.096
	GM (95% CI)	1.59 (1.51–1.68)	1.21 (1.14–1.29)	1.15 (1.08–1.22)	<0.001

Smoking categories were based on current smoking status at the time of the survey; non-smokers included never and former smokers. Moderate and heavy paternal smoking were defined as <20 and ≥20 cigarettes/day, respectively. Urinary cotinine concentrations are presented as creatinine-adjusted geometric means (GMs) with 95% confidence intervals (CIs), estimated from a generalized linear model of log-transformed cotinine with log-transformed urinary creatinine included as a covariate. Group differences were assessed using Pearson’s chi-squared tests for categorical variables; the p-value for the GM row was obtained from the Type 3 test for age group in the corresponding generalized linear model. Analyses used unweighted pooled data and are not nationally representative. CI: confidence interval. GM: geometric mean. KoNEHS: Korean National Environmental Health Survey. KRW: Korean Won. LOD: limit of detection. SHS: secondhand smoke.

Parent-reported SHS exposure (≥1 time/week) differed by age group (p<0.001), ranging from 9.5% in children aged 3–5 years to 15.6% in those aged 9–11 years. Between Cycle 3 and Cycle 4, parent-reported SHS exposure was lower in Cycle 4 than in Cycle 3 among those aged 3–5 years (14.7% vs 4.3%, p<0.001) and those aged 6–8 years (13.8% vs 7.4%, p=0.005). In contrast, the difference among those aged 9–11 years was not statistically significant (17.4% vs 13.6%, p=0.159) (Supplementary file Table S3).

Detectable urinary cotinine was observed in more than 84% of children across all age groups. The creatinine-adjusted geometric mean (GM) urinary cotinine concentration decreased significantly with increasing age (1.59, 1.21, and 1.15 ng/mL in the 3–5, 6–8, and 9–11 years groups, respectively; p<0.001). Between cycles, the proportion with cotinine ≥3 ng/mL was higher in Cycle 4 across all age groups (Cycle 3 vs Cycle 4: 3–5 years, 14.2% vs 30.1%; 6–8 years, 19.1% vs 30.6%; 9–11 years, 21.0% vs 33.8%; all p<0.001), and creatinine-adjusted GM cotinine concentrations were also higher in Cycle 4 in all age groups (all p<0.001) (Supplementary file Table S3). Among those aged 3–5 years surveyed in 2020, the proportion spending ≥19 hours/day at home on weekends increased markedly between cycles (34.5% vs 61.4%, p<0.001), whereas the corresponding proportions showed no comparable between-cycle increases among the older children surveyed in 2019.

### Household smoking status and urinary cotinine by age group

**Figure 2:**
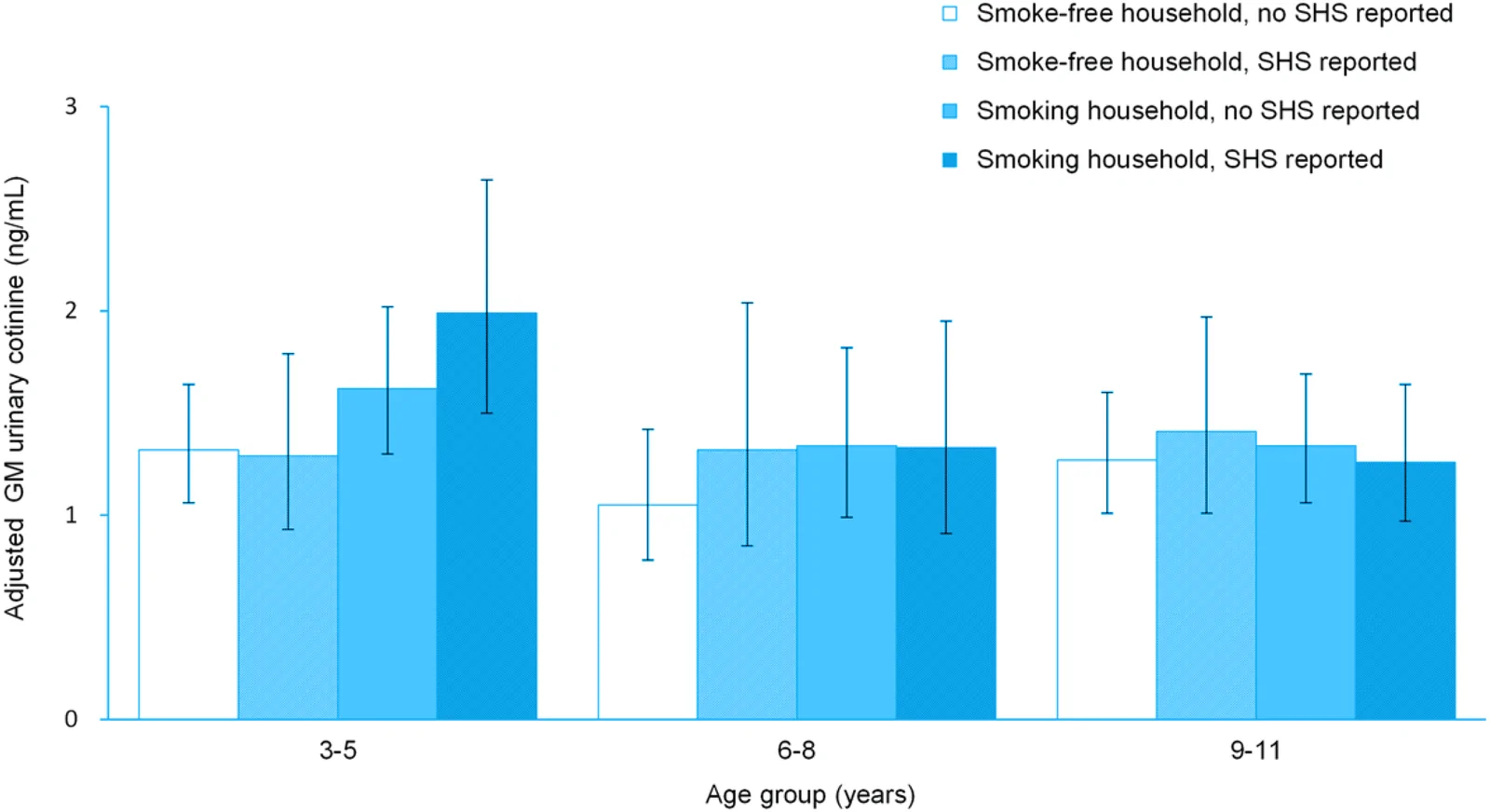
Multivariable-adjusted geometric mean (GM) urinary cotinine concentrations by household smoking status and parent-reported SHS exposure

Figure 2 shows multivariable-adjusted geometric mean (GM) urinary cotinine concentrations by household smoking status and parent-reported SHS exposure. Among those aged 3–5 years, those in the smoking household group without parent-reported SHS exposure had significantly higher adjusted GM cotinine concentrations than those in the smoke-free household group without parent-reported SHS exposure (GM: 1.62 vs 1.32 ng/mL; p<0.001), and those in the smoking household group with parent-reported SHS exposure had the highest concentrations (GM: 1.99 ng/mL; p<0.001). A similar pattern was observed among those aged 6–8 years in the smoking household group without parent-reported SHS exposure (GM: 1.34 vs 1.05 ng/mL; p=0.001).

Among those aged 9–11 years, however, neither those in the smoking household group without parent-reported SHS exposure (p=0.452) nor those in the smoking household group with parent-reported SHS exposure (p=0.902) differed significantly from those in the smoke-free household group without parent-reported SHS exposure.

In analyses stratified by household smoking status (Supplementary file Table S1), cotinine concentrations were significantly higher in those aged 3–5 years (β=0.47, p<0.001) and 6–8 years (β=0.16, p=0.014) than in those aged 9–11 years within smoking households. Cycle 4 was associated with higher urinary cotinine than Cycle 3 in both smoke-free (β=0.48, p<0.001) and smoking households (β=0.43, p<0.001).

### Concordance and correlates of questionnaire-negative/biomarker-positive discordance

Concordance between parent-reported SHS exposure and urinary cotinine-based classifications across three thresholds is shown in Table 2. Across all thresholds and age groups, concordance was negligible (κ range: -0.021–0.008), and McNemar’s test was statistically significant in 11 of 12 strata, indicating marked asymmetry toward questionnaire-negative/biomarker-positive discordance. Results at the ≥3 ng/mL threshold are emphasized because this threshold exceeded both cycle-specific limits of detection (Cycle 3: 0.3 ng/mL; Cycle 4: 0.2 ng/mL).

**Table 2:** Concordance between parent-reported secondhand smoke exposure and urinary cotinine-based classifications at three thresholds among children aged 3–11 years in a cross-sectional secondary analysis of KoNEHS Cycles 3 and 4, South Korea, 2016–2017 and 2019–2020 (N=2619)

*Urinary cotinine*	*Sensitivity (%)*	*Specificity (%)*	*PPV (%)*	*NPV (%)*	*κ (95% CI)*	*McNemar p*
≥**LOD**						
All	11.8	89.0	87.9	13.0	0.002 (-0.008–0.013)	<0.001
3–5 years	9.3	88.9	85.7	12.0	-0.005 (-0.020–0.010)	<0.001
6–8 years	11.3	91.2	87.8	15.5	0.008 (-0.011–0.027)	<0.001
9–11 years	15.9	86.4	89.9	11.8	0.006 (-0.015–0.027)	<0.001
≥**3 ng/mL**						
All	11.5	88.3	23.9	75.8	-0.002 (-0.037–0.032)	<0.001
3–5 years	9.0	90.3	21.0	77.7	-0.009 (-0.060–0.043)	<0.001
6–8 years	9.8	88.8	22.0	75.4	-0.017 (-0.079–0.045)	<0.001
9–11 years	16.1	84.5	27.7	73.2	0.007 (-0.060–0.075)	<0.001
≥**5 ng/mL**						
All	10.1	88.2	6.5	92.3	-0.014 (-0.049–0.021)	<0.001
3–5 years	7.1	90.3	4.8	93.5	-0.021 (-0.072–0.031)	0.006
6–8 years	9.8	89.0	7.3	91.8	-0.010 (-0.078–0.058)	0.067
9–11 years	13.2	84.1	7.6	90.8	-0.020 (-0.083–0.044)	<0.001

Biomarker positivity was defined at three urinary cotinine thresholds: ≥LOD, ≥3 ng/mL, and ≥5 ng/mL. Parent-reported SHS exposure was treated as the index classification. Sensitivity was defined as the proportion of children meeting a urinary cotinine threshold who were classified as exposed by parent report, whereas specificity was the proportion below the threshold who were classified as unexposed by parent report. PPV and NPV were calculated from the same cross-classification. The prespecified urinary cotinine thresholds served as operational comparison criteria and were not treated as gold-standard definitions of SHS exposure. Concordance was assessed using Cohen’s κ, and directional asymmetry in discordant pairs was evaluated using McNemar’s test. Analyses used unweighted pooled data from KoNEHS Cycles 3 (2016–2017) and 4 (2019–2020) and are not nationally representative. CI: confidence interval. κ: Cohen’s kappa. KoNEHS: Korean National Environmental Health Survey. LOD: limit of detection. NPV: negative predictive value. PPV: positive predictive value. SHS: secondhand smoke.

At this threshold, the sensitivity of parent-reported SHS exposure was consistently low across age groups (3–5 years, 9.0%; 6–8 years, 9.8%; 9–11 years, 16.1%), with correspondingly high questionnaire-negative proportions among biomarker-positive children (91.0%, 90.2%, and 83.9%, respectively); specificity, however, remained high (90.3%, 88.8%, and 84.5%). Analyses at ≥LOD and ≥5 ng/mL yielded directionally consistent findings (Table 2).

To identify correlates of questionnaire-negative/biomarker-positive discordance, multivariable logistic regression was conducted among biomarker-positive children (Table 3). At ≥LOD, odds of questionnaire-negative/biomarker-positive discordance were higher in those aged 3–5 years than in 9–11 years (AOR=1.64; 95% CI: 1.20–2.24, p=0.002); the estimate for those aged 6–8 years did not reach significance (AOR=1.39, 95% CI: 1.00-1.95, p=0.051).

**Table 3:** Adjusted odds ratios for questionnaire-negative/biomarker-positive discordance at two urinary cotinine thresholds among biomarker-positive children aged 3–11 years in a cross-sectional secondary analysis of KoNEHS Cycles 3 and 4, South Korea, 2016–2017 and 2019–2020 (≥LOD: N=2282; ≥3 ng/mL: N=633)

*Variables*	*Cotinine ≥LOD*		*Cotinine ≥3 ng/mL*	
	*AOR (95% CI)*	*p*	*AOR (95% CI)*	*p*
**Age group** (ref: 9–11 years)				
3–5 years	1.64 (1.20–2.24)	0.002	1.95 (1.03–3.67)	0.039
6–8 years	1.39 (1.00–1.95)	0.051	1.81 (0.92–3.55)	0.084
**Survey cycle** (Cycle 4 vs Cycle 3)				
Overall	2.09 (1.59–2.75)	<0.001	3.52 (2.01–6.15)	<0.001
**Age-stratified estimates**				
3–5 years	3.97 (2.38–6.61)	<0.001	5.70 (1.92–16.91)	0.002
6–8 years	2.12 (1.25–3.59)	0.005	2.94 (0.96–8.97)	0.059
9–11 years	1.24 (0.81–1.92)	0.328	3.44 (1.45–8.15)	0.005
**Sex** (ref: Female)				
Male	0.93 (0.71–1.21)	0.565	0.58 (0.33–0.99)	0.048
**Paternal education** (ref: College or higher)				
High school or lower	0.46 (0.33–0.63)	<0.001	0.40 (0.22–0.73)	0.003
**Maternal education** (ref: College or higher)				
High school or lower	0.94 (0.68–1.29)	0.688	1.03 (0.56–1.91)	0.914
**Paternal smoking** (ref: Non-smoker)				
Moderate smoker	0.53 (0.39–0.71)	<0.001	0.41 (0.22–0.76)	0.005
Heavy smoker	0.44 (0.30–0.63)	<0.001	0.34 (0.16–0.71)	0.004
**Maternal smoking** (ref: Non-smoker)				
Smoker	0.35 (0.14–0.85)	0.021	0.41 (0.09–1.80)	0.236

Questionnaire-negative/biomarker-positive discordance was defined as the absence of parent-reported SHS exposure despite urinary cotinine at or above the prespecified threshold. Logistic regression was restricted to biomarker-positive children (≥LOD, N=2282; ≥3 ng/mL, N=633). Overall models were adjusted for age group, survey cycle, sex, education of both parents, and paternal and maternal smoking status. Parental smoking categories were based on current smoking at the time of the survey, with never and former smokers classified as non-smokers. Age-group-specific survey-cycle estimates were obtained from models fitted separately within each age group and adjusted for the remaining covariates. The ≥5 ng/mL threshold was excluded because of sparse cells and the resulting imprecision of the estimates. Analyses were conducted using unweighted pooled data from KoNEHS Cycles 3 and 4 and are not nationally representative. Cycle 3 was conducted in 2016–2017 and Cycle 4 in 2019–2020. Within Cycle 4, children aged 6–8 and 9–11 years were surveyed in 2019, whereas children aged 3–5 years were surveyed in 2020. AOR: adjusted odds ratio. CI: confidence interval. KoNEHS: Korean National Environmental Health Survey. LOD: limit of detection. SHS: secondhand smoke.

Survey cycle was also associated with discordance: Cycle 4 was associated with more than twice the odds of questionnaire-negative/biomarker-positive discordance overall at ≥LOD (AOR=2.09; 95% CI: 1.59–2.75, p<0.001), with age-group-specific estimates largest among children aged 3–5 years and progressively smaller in the older age groups (3–5 years: AOR=3.97; 95% CI: 2.38–6.61; 6–8 years: AOR=2.12; 95% CI: 1.25–3.59; 9–11 years: AOR=1.24; 95% CI: 0.81–1.92). At ≥3 ng/mL, Cycle 4 was associated with higher odds of discordance than Cycle 3 (AOR=3.52; 95% CI: 2.01–6.15, p<0.001).

A paternal education level of high school or lower was associated with lower odds of questionnaire-negative/biomarker-positive discordance (AOR=0.46; 95% CI: 0.33–0.63, p<0.001).

Paternal smoking intensity was inversely associated with questionnaire-negative/biomarker-positive discordance (moderate vs non-smoker: AOR=0.53; 95% CI: 0.39–0.71; heavy vs non-smoker: AOR=0.44; 95% CI: 0.30–0.63; both p<0.001).

### Age-stratified associations between paternal smoking and urinary cotinine

In those aged 3–5 years, paternal smoking was associated with higher cotinine concentrations (adjusted GMs: 1.38, 1.70, and 1.90 ng/mL across the non-smoker, moderate-smoker, and heavy-smoker categories, respectively; β=0.21 and β=0.32 for moderate and heavy smoking, respectively, both p<0.001). In those aged 6–8 years, a positive gradient was also observed (adjusted GMs: 0.58, 0.68, and 0.85 ng/mL; moderate smoking, β=0.17, p=0.027; heavy smoking, β=0.39, p<0.001).

Among those aged 9–11 years, however, adjusted GM cotinine values were comparable across paternal smoking categories (1.58, 1.60, and 1.60 ng/mL), and the coefficients for moderate and heavy paternal smoking were both near null (β=0.01; both p>0.88), despite similar paternal smoking prevalence across age groups (Table 1). Additional adjustment for time-activity variables did not materially alter the age-stratified paternal smoking coefficients (all absolute changes <0.01).

Compared with a vigorous physical activity frequency of 0–2 times/week, daily vigorous physical activity was positively associated with urinary cotinine in those aged 6–8 years (β=0.31; 95% CI: 0.08–0.55, p=0.008) and 9–11 years (β=0.32; 95% CI: 0.10–0.53, p=0.005), but not in those aged 3–5 years. Cycle-specific weighted sensitivity analyses (Supplementary file Table S5) yielded estimates that were directionally consistent with the corresponding unweighted estimates.

Exploratory interaction models (Supplementary file Table S4) suggested that the cotinine contrast between children aged 3–5 and 9–11 years was greater in Cycle 4, whereas the association of heavy paternal smoking was attenuated in Cycle 4; given multiple comparisons, these findings were interpreted descriptively.

## DISCUSSION

This study found consistently low concordance between parent-reported SHS exposure and urinary cotinine-based classifications across two independent cross-sectional KoNEHS cycles. Questionnaire-negative/biomarker-positive discordance predominated and was more frequent among younger children, children whose fathers had a higher level of education, and children surveyed in Cycle 4. Among the two younger age groups, children in smoking households also had higher urinary cotinine concentrations in the absence of parent-reported SHS exposure.

The association between paternal smoking and urinary cotinine was evident in the two younger age groups but was not statistically significant in the oldest age group. Together, these patterns suggest that parent-reported exposure events and urinary cotinine provide related but non-interchangeable information and that discordance between these measures varies across developmental and survey contexts.

Several mechanisms may contribute to the observed discordance. Parent-completed questionnaires capture parent-recognized SHS exposure events, whereas urinary cotinine reflects recent internal nicotine burden, regardless of whether the parent recognized the exposure source or event. These measures therefore represent related but distinct exposure constructs and are not directly interchangeable, even under ideal reporting conditions. Behavioral or product-related shifts that reduce the perceptibility of household nicotine transfer have also been proposed, although this explanation remains hypothetical because behavioral visibility was not directly measured^13-15^.

### Concordance and directional discordance

The negligible concordance (κ≈0), together with the asymmetry of discordant pairs, indicates a directional pattern of discordance rather than symmetric disagreement. This pattern may reflect both reporting bias and construct divergence between parent-reported SHS exposure and urinary cotinine-based classifications^6,27^.

Distinguishing between these mechanisms has implications for exposure surveillance: reporting-related discordance may be reduced through improved parent reporting, whereas construct divergence supports the complementary use of urinary cotinine alongside questionnaire-based assessment. Low sensitivity and poor concordance are consistent with prior discordance studies^6,8,25^. In Japan, the prevalence of SHS exposure classified using urinary cotinine declined substantially from 2011 to 2021, alongside reductions in parental tobacco use^28^. This contrasts with the higher urinary cotinine concentrations observed in Cycle 4 of the present study, despite lower parent-reported exposure.

### Education, reporting behavior, and smoking intensity

Questionnaire-negative/biomarker-positive discordance was more likely among children whose fathers had a higher level of education, a pattern consistent with socially patterned reporting. Paternal education was not associated with urinary cotinine in any age group (Supplementary file Table S2), suggesting that the discordance gradient was more consistent with reporting differences than with marked differences in cotinine concentrations across educational strata^6,7^. Higher level of paternal education may be associated with greater awareness of social norms discouraging smoking near children, which could influence how SHS exposure is recognized or reported.

The inverse association between paternal smoking intensity and the odds of questionnaire-negative/biomarker-positive discordance is consistent with greater exposure perceptibility in heavy-smoking households, where smoking-related exposure may be more noticeable and therefore more likely to be reported. This pattern suggests that exposure visibility, rather than exposure magnitude alone, may influence the relationship between parent reports and urinary cotinine-based classifications.

### Between-Cycle differences

Between-Cycle comparisons reflect two independent cross-sectional samples and should not be interpreted as longitudinal trends. Comparisons are anchored at the ≥3 ng/mL threshold to minimize the influence of differing detection limits (Cycle 3: 0.3 ng/mL; Cycle 4: 0.2 ng/mL). The consistency of this divergence across thresholds and age groups suggests evolving exposure-reporting dynamics; however, independent sampling frames and the partial overlap of Cycle 4 with the COVID-19 pandemic among those aged 3–5 years limit attribution of the observed differences to temporal changes in exposure or reporting.

Several factors may account for this between-cycle divergence. Strengthening indoor smoking restrictions may have shifted smoking toward less conspicuous patterns, reducing parent-perceived exposure without eliminating nicotine transfer^13,15^. The increasing use of heated tobacco products after 2017 may also have altered parental perceptions of what constitutes SHS; however, product type was not measured, and cotinine cannot distinguish among cigarettes, heated tobacco products, and e-cigarettes as nicotine sources^14,19^.

Pandemic-related behavioral changes may also have influenced exposure opportunity among preschool-aged children^20^. However, among children aged 6–8 and 9–11 years who were surveyed in 2019 before the COVID-19 pandemic, the estimated odds of questionnaire-negative/biomarker-positive discordance at the ≥3 ng/mL threshold and the proportions meeting this threshold were higher in Cycle 4 than in Cycle 3 (Table 3; Supplementary file Table S3). These findings suggest that pandemic-related confinement alone is unlikely to explain the between-cycle divergence fully.

### Age-dependent exposure context

The inverse age gradient in urinary cotinine concentrations is consistent with younger children spending more time in the home environment, where opportunities for smoking-related exposure may be greater^4,22,29,30^. Among children aged 3–5 and 6–8 years, those in smoking households had higher urinary cotinine concentrations even in the absence of parent-reported SHS exposure (Figure 2)^31^, a pattern consistent with reporting differences or indirect exposure pathways, including thirdhand smoke residue and other environmental nicotine transfer^13,32^. This pattern was not evident among children aged 9–11 years.

The attenuated associations among children aged 9–11 years do not necessarily indicate the absence of nicotine exposure. Older children may spend more time outside the home, weakening associations between household smoking indicators and urinary cotinine. The positive association between daily vigorous physical activity and urinary cotinine in this age group may reflect broader activity-related exposure contexts, although exposure location and source were not measured. Rather than contradicting the discordance findings, these patterns may indicate that household smoking indicators become less dominant with age.

### Strengths and limitations

Strengths include a nationwide biomonitoring dataset spanning two independent cross-sectional survey cycles, objective quantification of urinary cotinine by GC-MS with external quality assurance, evaluation of multiple prespecified cotinine thresholds, and formal assessment of the direction of discordance using McNemar’s test.

Limitations include the cross-sectional design and between-cycle heterogeneity, including differing LODs (0.3 vs 0.2 ng/mL), independent sampling frames, and partial overlap with the COVID-19 pandemic; these features limit causal interpretation and attribution of between-cycle differences to temporal changes. Tobacco product type was not measured, and urinary cotinine does not distinguish among nicotine sources^14^. Urinary cotinine reflected recent internal nicotine burden from a single spot urine specimen, whereas the questionnaire assessed frequency using weekly categories rather than a specified recall window; this difference in temporal framing may have contributed to discordance. Parental smoking and children’s SHS exposure were parent-reported and may be subject to reporting bias^6^. Ambient nicotine concentrations and specific exposure settings or sources were not assessed. Reporting bias and construct divergence could not be empirically distinguished within the present design; additional source-specific environmental measurements and repeated biomarker assessments would be needed to separate these mechanisms.

The patterned discordance across age, education, and survey cycle suggests that questionnaire-based classification alone may not fully characterize subgroup differences in internal nicotine burden. Complementary use of urinary cotinine alongside parent-reported SHS assessment may therefore be useful in pediatric research, particularly in subgroups with greater discordance.

## CONCLUSIONS

Across two independent cross-sectional KoNEHS cycles, parent-reported SHS exposure showed negligible agreement with urinary cotinine-based classifications, with questionnaire-negative/biomarker-positive discordance more frequent among younger children and in Cycle 4. The pattern of discordance by age, paternal education level, smoking intensity, and survey cycle is consistent with reporting processes, construct divergence, and potentially changing exposure environments rather than random measurement error alone. These findings indicate that parent-reported SHS exposure and urinary cotinine provide related but non-interchangeable information; urinary cotinine may complement questionnaire-based assessment when characterizing children’s internal nicotine burden.

## Supplementary Material



## Data Availability

KoNEHS microdata are available to eligible researchers through the Environmental Health Information System upon application and approval^33^. The analysis code is available from the corresponding author upon reasonable request.

## References

[1] Kaur J, Upendra S, Barde S. “Inhaling hazards, exhaling insights: a systematic review unveiling the silent health impacts of secondhand smoke pollution on children and adolescents. Int J Environ Health Res. 2024;34(12):4059-4073. doi:10.1080/09603123.2024.2337837 38576330

[2] Oberg M, Jaakkola MS, Woodward A, Peruga A, Pruss-Ustun A. Worldwide burden of disease from exposure to second-hand smoke: a retrospective analysis of data from 192 countries. Lancet. 2011;377(9760):139-46. doi:10.1016/S0140-6736(10)61388-8 21112082

[3] Flor LS, Anderson JA, Ahmad N, et al. Health effects associated with exposure to secondhand smoke: a burden of proof study. Nat Med. 2024;30(1):149-167. doi:10.1038/s41591-023-02743-4 38195750 PMC10803272

[4] Seong MW, Moon JS, Hwang JH, et al. Preschool children and their mothers are more exposed to paternal smoking at home than school children and their mothers. Clin Chim Acta. 2010;411(1-2):72-76. doi:10.1016/j.cca.2009.10.006 19833114

[5] Ahmed F, Gawde N, Parasuraman S. Prevalence, sources, and correlates of second-hand smoke exposure among non-smoking pregnant women in India. J Prev Med Public Health. 2025;58(2):136-145. doi:10.3961/jpmph.24.278 39901756 PMC11986596

[6] Mourino N, Perez-Rios M, Santiago-Perez MI, Lanphear B, Yolton K, Braun JM. Secondhand tobacco smoke exposure among children under 5 years old: questionnaires versus cotinine biomarkers: a cohort study. BMJ Open. 2021;11(6):e044829. doi:10.1136/bmjopen-2020-044829 PMC8240561 34183339

[7] Sim B, Park MB. Exposure to secondhand smoke: inconsistency between self-response and urine cotinine biomarker based on Korean national data during 2009-2018. Int J Environ Res Public Health. 2021;18(17):9284. doi:10.3390/ijerph18179284 34501873 PMC8431172

[8] Dempsey DA, Meyers MJ, Oh SS, et al. Determination of tobacco smoke exposure by plasma cotinine levels in infants and children attending urban public hospital clinics. Arch Pediatr Adolesc Med. 2012;166(9):851-856. doi:10.1001/archpediatrics.2012.170 22566513 PMC3997061

[9] Kim J, Lee K. Characterization of urinary cotinine in non-smoking residents in smoke-free homes in the Korean National Environmental Health Survey (KoNEHS). BMC Public Health. 2016;16:538. doi:10.1186/s12889-016-3212-9 27401105 PMC4939825

[10] Jeong SH, Jang BN, Kang SH, Joo JH, Park EC. Association between parents’ smoking status and tobacco exposure in school-age children: assessment using major urine biomarkers. Sci Rep. 2021;11(1):4536. doi:10.1038/s41598-021-84017-y 33633242 PMC7907361

[11] Aquilina NJ, Jacob P, 3rd, Benowitz NL, Fsadni P, Montefort S. Secondhand smoke exposure in school children in Malta assessed through urinary biomarkers. Environ Res. 2022;204(Pt D):112405. doi:10.1016/j.envres.2021.112405 34822856 PMC9119146

[12] Campo L, Boniardi L, Polledri E, Longhi F, Scuffi C, Fustinoni S. Smoking habit in parents and exposure to environmental tobacco smoke in elementary school children of Milan. Sci Total Environ. 2021;796:148891. doi:10.1016/j.scitotenv.2021.148891 34274675

[13] Matt GE, Greiner L, Record RA, et al. Policy-relevant differences between secondhand and thirdhand smoke: strengthening protections from involuntary exposure to tobacco smoke pollutants. Tob Control. 2024;33(6):798-806. doi:10.1136/tc-2023-057971 37263783 PMC11503167

[14] Tattan-Birch H, Brown J, Jackson SE, Jarvis MJ, Shahab L. Secondhand nicotine absorption from e-cigarette vapor vs tobacco smoke in children. JAMA Netw Open. 2024;7(7):e2421246. doi:10.1001/jamanetworkopen.2024.21246 38990571 PMC11240186

[15] Torres S, Merino C, Paton B, Correig X, Ramirez N. Biomarkers of exposure to secondhand and thirdhand tobacco smoke: recent advances and future perspectives. Int J Environ Res Public Health. 2018;15(12):2693. doi:10.3390/ijerph15122693 30501044 PMC6313747

[16] Lee B, Kwon M, Park AH, Kim H. Tobacco use trends in South Korea, 2013-2023: Persistent disparities and emerging challenges in a repeated cross-sectional study. Tob Induc Dis. 2026;24(February):14. doi:10.18332/tid/215655 PMC12879552 41657541

[17] Park JE, Jeong WM, Choi YJ, Kim SY, Yeob KE, Park JH. Tobacco use in Korea: current epidemiology and public health issues. J Korean Med Sci. 2024;39(45):e328. doi:10.3346/jkms.2024.39.e328 39592131 PMC11596473

[18] Lee CM. The impact of heated tobacco products on smoking cessation, tobacco use, and tobacco sales in South Korea. Korean J Fam Med. 2020;41(5):273-281. doi:10.4082/kjfm.20.0140 32961046 PMC7509116

[19] Kim SH, Cho HJ. Prevalence and correlates of current use of heated tobacco products among a nationally representative sample of Korean adults: results from a cross-sectional study. Tob Induc Dis. 2020;18(August):66. doi:10.18332/tid/125232 32818029 PMC7425751

[20] Osinibi M, Gupta A, Harman K, Bossley CJ. Passive tobacco smoke in children and young people during the COVID-19 pandemic. Lancet Respir Med. 2021;9(7):693-694. doi:10.1016/S2213-2600(21)00231-9 34029535 PMC9764366

[21] Barr DB, Wilder LC, Caudill SP, Gonzalez AJ, Needham LL, Pirkle JL. Urinary creatinine concentrations in the U.S. population: implications for urinary biologic monitoring measurements. Environ Health Perspect. 2005;113(2):192-200. doi:10.1289/ehp.7337 15687057 PMC1277864

[22] Park MB, Ranabhat CL. Effect of parental smoking on their children’s urine cotinine level in Korea: a population-based study. PLoS One. 2021;16(4):e0248013. doi:10.1371/journal.pone.0248013 33857161 PMC8049314

[23] Hahn D, Schmied-Tobies M, Rucic E, et al. Urinary cotinine and exposure to passive smoke in children and adolescents in Germany - Human biomonitoring results of the German Environmental Survey 2014-2017 (GerES V). Environ Res. 2023;216(Pt 1):114320. doi:10.1016/j.envres.2022.114320 36100102

[24] Mourino N, Ruano-Raviña A, Varela Lema L, et al. Serum cotinine cut-points for secondhand smoke exposure assessment in children under 5 years: a systemic review. PLoS One. 2022;17(5):e0267319. doi:10.1371/journal.pone.0267319 35511766 PMC9070924

[25] Wilkinson JD, Arheart KL, Lee DJ. Accuracy of parental reporting of secondhand smoke exposure: the national health and nutrition examination survey III. Nicotine Tob Res. 2006;8(4):591-7. doi:10.1080/14622200600790021 16920657

[26] SAS Institute Inc. SAS/STAT 9.4 User’s Guide. Cary, NC: SAS Institute Inc.; 2013.

[27] Florescu A, Ferrence R, Einarson T, Selby P, Soldin O, Koren G. Methods for quantification of exposure to cigarette smoking and environmental tobacco smoke: focus on developmental toxicology. Ther Drug Monit. 2009;31(1):14-30. doi:10.1097/FTD.0b013e3181957a3b 19125149 PMC3644554

[28] Tamada Y, Takeuchi K, Tabuchi T. Secondhand tobacco exposure assessed using urinary cotinine among 10-year-old children in Japan: an 11-year repeated cross-sectional study. Nicotine Tob Res. 2025;27(3):534-541. doi:10.1093/ntr/ntae220 39297512 PMC11847774

[29] Tung KY, Wu KY, Tsai CH, et al. Association of time-location patterns with urinary cotinine among asthmatic children under household environmental tobacco smoke exposure. Environ Res. 2013;124:7-12. doi:10.1016/j.envres.2013.03.002 23623351

[30] Kim D, Guak S, Lee K. Temporal trend of microenvironmental time-activity patterns of the Seoul population from 2004 to 2022 and its potential impact on exposure assessment. J Expo Sci Environ Epidemiol. 2025;35(2):315-324. doi:10.1038/s41370-024-00662-1 38548930 PMC12009733

[31] Park MB. Living with parents who smoke predicts levels of toxicant exposure in children. Sci Rep. 2020;10(1):11173. doi:10.1038/s41598-020-66920-y 32636401 PMC7341805

[32] Merianos AL, Stone TM, Jandarov RA, Mahabee-Gittens EM, Choi K. Sources of tobacco smoke exposure and their associations with serum cotinine levels among US children and adolescents. Nicotine Tob Res. 2023;25(5):1004-1013. doi:10.1093/ntr/ntac293 36567673 PMC10077940

[33] National Institute of Environmental Research. Korean National Environmental Health Survey: microdata request. Environmental Health Information System.

