# Supplementary material for: Biomarker–questionnaire discordance in secondhand smoke exposure among Korean children: A secondary analysis of the Korean National Environmental Health Survey, 2016-2017 and 2019-2020
Source: Tob Induc Dis. 2026 Sep 4;24:10.18332/tid/226880. doi: 10.18332/tid/226880 (PMC13544052; doi:10.18332/tid/226880)
Supplement: Supplementary file 1 [file TID-24-155-s1.pdf]

Tobacco Induced Diseases

Supplementary file

© 2026 Lee H. J. and Cho S. I.

DOI:
10.18332/tid/226880

The content has been provided by the author(s) and has not been reviewed, verified, or endorsed by European Publishing. It may not have undergone peer review. The views, opinions, and recommendations expressed are solely those of the author(s) and do not necessarily reflect the position of European Publishing. European Publishing accepts no responsibility or liability for any consequences arising from the use of, or reliance on, this content.

Supplementary Table S1. Multivariable linear regression of log-transformed urinary cotinine stratified by household smoking status among children aged 3-11 years in a cross-sectional secondary analysis of KoNEHS Cycles 3 and 4, South Korea, 2016-2017 and 2019-2020 (smoke-free household: N=1489; smoking household: N=1130)

Variables	Smoke-free household (N=1489)		Smoking household (N=1130)	
	β (95% CI)	p	β (95% CI)	p
**Age group (ref: 9-11 years)**				
3-5 years	0.19 (0.08, 0.31)	0.001	0.47 (0.34, 0.59)	<0.001
6-8 years	-0.01 (-0.13, 0.11)	0.900	0.16 (0.03, 0.29)	0.014
**Survey cycle (ref: Cycle 3)**				
Cycle 4	0.48 (0.39, 0.57)	<0.001	0.43 (0.33, 0.53)	<0.001
**Maternal occupation (ref: Non-manual)**				
Service	0.03 (-0.13, 0.20)	0.720	0.22 (0.06, 0.39)	0.007
Manual	-0.11 (-0.35, 0.13)	0.380	0.22 (-0.01, 0.46)	0.063
Unemployed	-0.01 (-0.11, 0.09)	0.824	0.06 (-0.05, 0.17)	0.300
**Vigorous physical activity (ref: 0-2 times/week)**				
3-6 times/week	0.01 (-0.09, 0.10)	0.895	0.03 (-0.07, 0.14)	0.543
Daily (7 times/week)	0.17 (0.01, 0.33)	0.036	0.13 (-0.03, 0.30)	0.114
**Current allergic disease (ref: No)**				
Any	-0.12 (-0.21, -0.02)	0.019	-0.03 (-0.13, 0.07)	0.564
**Home ventilation frequency (ref: 1-3 times/week)**				
4-6 times/week	-0.13 (-0.25, -0.01)	0.037	-0.04 (-0.17, 0.09)	0.546
7 times/week	-0.04 (-0.17, 0.10)	0.596	0.06 (-0.08, 0.21)	0.383

Selected coefficients are shown. Values are β coefficients with 95% confidence intervals (CIs) from linear regression models of log-transformed urinary cotinine. Exponentiating β yields the corresponding geometric mean ratio (GMR). Age-group coefficients were estimated separately within smoke-free and smoking households from models adjusted for survey cycle, sex, vigorous physical activity, housing type, building age, household income, education and occupation of both parents, home ventilation frequency, current allergic disease, and log-transformed urinary creatinine. Paternal and maternal smoking variables were not included because household smoking status was derived from current paternal and maternal smoking. Smoke-free denotes no current parental smoker; smoking denotes at least one current parental smoker. Analyses used unweighted pooled data from KoNEHS Cycles 3 (2016-2017) and 4 (2019-2020) and are not nationally representative.
CI: confidence interval; GMR: geometric mean ratio; KoNEHS: Korean National Environmental Health Survey.

Supplementary Table S2. Age-stratified multivariable linear regression of log-transformed urinary cotinine among children aged 3-11 years in a cross-sectional secondary analysis of KoNEHS Cycles 3 and 4, South Korea, 2016-2017 and 2019-2020 (3-5 years: N=1104; 6-8 years: N=754; 9-11 years: N=761)

Variables	3-5 years (N=1104)		6-8 years (N=754)		9-11 years (N=761)	
	β (95% CI)	p	β (95% CI)	p	β (95% CI)	p
**Survey cycle (ref: Cycle 3)**						
Cycle 4	0.65 (0.55, 0.74)	<0.001	0.32 (0.19, 0.46)	<0.001	0.31 (0.19, 0.43)	<0.001
**Sex (ref: Female)**						
Male	0.08 (-0.02, 0.17)	0.119	-0.08 (-0.22, 0.05)	0.228	0.12 (0.00, 0.25)	0.056
**Housing type (ref: Detached)**						
Apartment	0.08 (-0.07, 0.24)	0.283	-0.07 (-0.27, 0.13)	0.501	0.06 (-0.11, 0.23)	0.484
Attached	0.13 (-0.09, 0.34)	0.260	-0.20 (-0.46, 0.06)	0.138	-0.09 (-0.33, 0.15)	0.469
**Building age (ref: <30 years)**						
≥30 years	0.25 (0.05, 0.44)	0.015	0.08 (-0.20, 0.37)	0.562	-0.09 (-0.33, 0.14)	0.447
**Household income (10000 KRW/month; ref: ≥700)**						
<300	-0.01 (-0.19, 0.16)	0.869	0.08 (-0.17, 0.33)	0.539	0.29 (0.07, 0.51)	0.010
300-700	0.01 (-0.12, 0.14)	0.870	0.01 (-0.18, 0.19)	0.944	-0.08 (-0.26, 0.09)	0.348
**Paternal education (ref: College or higher)**						
High school or lower	0.08 (-0.07, 0.22)	0.293	0.01 (-0.18, 0.20)	0.906	0.01 (-0.15, 0.18)	0.869
**Maternal education (ref: College or higher)**						
High school or lower	0.08 (-0.06, 0.22)	0.266	0.02 (-0.16, 0.21)	0.817	-0.06 (-0.22, 0.09)	0.439
**Paternal occupation (ref: Non-manual)**						
Service	0.08 (-0.06, 0.23)	0.256	-0.14 (-0.33, 0.06)	0.168	-0.02 (-0.20, 0.15)	0.784
Manual	0.17 (0.05, 0.29)	0.005	-0.08 (-0.25, 0.08)	0.311	0.03 (-0.12, 0.18)	0.734
Unemployed	-0.04 (-0.75, 0.68)	0.918	-0.89 (-1.96, 0.19)	0.106	-0.75 (-1.39, -0.12)	0.021
**Maternal occupation (ref: Non-manual)**						
Service	0.06 (-0.13, 0.24)	0.544	0.00 (-0.24, 0.24)	0.986	0.21 (0.02, 0.40)	0.032
Manual	-0.08 (-0.33, 0.17)	0.517	0.25 (-0.10, 0.59)	0.159	0.03 (-0.27, 0.34)	0.823
Unemployed	0.06 (-0.05, 0.17)	0.261	-0.04 (-0.19, 0.10)	0.560	-0.04 (-0.17, 0.10)	0.592
**Paternal smoking (ref: Non-smoker)**						
Moderate smoker	0.21 (0.10, 0.31)	<0.001	0.17 (0.02, 0.31)	0.027	0.01 (-0.13, 0.15)	0.887
Heavy smoker	0.32 (0.16, 0.47)	<0.001	0.39 (0.18, 0.61)	<0.001	0.01 (-0.18, 0.20)	0.914
**Maternal smoking (ref: Non-smoker)**						
Smoker	0.11 (-0.42, 0.65)	0.674	-1.22 (-2.50, 0.06)	0.061	0.43 (-0.02, 0.89)	0.063
**Vigorous physical activity (ref: 0-2 times/week)**						
3-6 times/week	0.00 (-0.10, 0.10)	0.996	0.06 (-0.09, 0.21)	0.411	0.03 (-0.10, 0.16)	0.644
Daily (7 times/week)	0.00 (-0.17, 0.17)	0.998	0.31 (0.08, 0.55)	0.008	0.32 (0.10, 0.53)	0.005
**Current allergic disease (ref: No)**						

Variables	3-5 years (N=1104)		6-8 years (N=754)		9-11 years (N=761)	
Any	-0.06 (-0.17, 0.04)	0.250	-0.15 (-0.29, -0.01)	0.033	-0.05 (-0.18, 0.07)	0.393
**Home ventilation frequency (ref: 1-3 times/week)**						
4-6 times/week	-0.06 (-0.19, 0.07)	0.365	-0.05 (-0.23, 0.12)	0.556	-0.11 (-0.28, 0.05)	0.172
7 times/week	0.00 (-0.13, 0.14)	0.961	-0.01 (-0.21, 0.20)	0.957	0.05 (-0.15, 0.24)	0.634

Values are β coefficients with 95% confidence intervals (CIs) from linear regression models of log-transformed urinary cotinine. Exponentiating β yields the corresponding geometric mean ratio (GMR). Models were adjusted for survey cycle, sex, housing type, building age, household income, education and occupation of both parents, paternal smoking, maternal smoking, vigorous physical activity, current allergic disease, home ventilation frequency, and log-transformed urinary creatinine. Age-stratified models were not designed to test interaction; the estimates are interpreted descriptively. Smoking categories were based on current smoking status at the time of the survey; non-smokers included never and former smokers. Analyses used unweighted pooled data from KoNEHS Cycles 3 (2016-2017) and 4 (2019-2020) and are not nationally representative.
CI: confidence interval; GMR: geometric mean ratio; KoNEHS: Korean National Environmental Health Survey; KRW: Korean won.

Supplementary Table S3. Characteristics of children aged 3-11 years by age group and survey cycle in a cross-sectional secondary analysis of KoNEHS Cycles 3 and 4, South Korea, 2016-2017 and 2019-2020 (N=2618)

**Variables**	**3-5 years**			**6-8 years**			**9-11 years**		
	**Cycle 3 (N=550)**	**Cycle 4 (N=554)**	**p**	**Cycle 3 (N=413)**	**Cycle 4 (N=340)**	**p**	**Cycle 3 (N=409)**	**Cycle 4 (N=352)**	**p**
	**n (%)**	**n (%)**		**n (%)**	**n (%)**		**n (%)**	**n (%)**	
**Paternal smoking**									
Non-smoker	307 (55.8)	333 (60.1)	0.005	234 (56.7)	188 (55.3)	0.146	220 (53.8)	209 (59.4)	0.137
Moderate smoker (<20 cigarettes/day)	164 (29.8)	176 (31.8)		122 (29.5)	118 (34.7)		128 (31.3)	106 (30.1)	
Heavy smoker (≥20 cigarettes/day)	79 (14.4)	45 (8.1)		57 (13.8)	34 (10.0)		61 (14.9)	37 (10.5)	
Cigarettes/day among paternal smokers, mean (SE)	13.5 (0.5)	11.7 (0.4)	0.005	13.3 (0.5)	11.9 (0.5)	0.050	13.7 (0.5)	13.7 (0.7)	0.941
**Maternal smoking**									
Non-smoker	545 (99.1)	550 (99.3)	0.730	412 (99.8)	339 (99.7)	0.890	401 (98.0)	346 (98.3)	0.797
Smoker	5 (0.9)	4 (0.7)		1 (0.2)	1 (0.3)		8 (2.0)	6 (1.7)	
**Household smoking**									
Smoke-free	307 (55.8)	333 (60.1)	0.149	234 (56.7)	187 (55.0)	0.648	220 (53.8)	208 (59.1)	0.142
Smoking	243 (44.2)	221 (39.9)		179 (43.3)	153 (45.0)		189 (46.2)	144 (40.9)	
**Weekday indoor time at home (ref: <15 h/day)**									
≥15 h/day	215 (39.1)	329 (59.4)	<0.001	137 (33.2)	122 (35.9)	0.436	93 (22.7)	83 (23.6)	0.784
**Weekday kindergarten/school time (ref: <7 h/day)**									
≥7 h/day	371 (67.5)	334 (60.3)	0.013	97 (23.5)	56 (16.5)	0.017	137 (33.5)	109 (31.0)	0.457
**Weekday indoor time at other locations (ref: <2 h/day)**									
≥2 h/day	63 (11.5)	60 (10.8)	0.742	224 (54.2)	217 (63.8)	0.008	236 (57.7)	233 (66.2)	0.016
**Weekend indoor time at home (ref: <19 h/day)**									
≥19 h/day	190 (34.5)	340 (61.4)	<0.001	130 (31.5)	110 (32.4)	0.797	151 (36.9)	129 (36.6)	0.938
**Weekend indoor time at other locations (ref: <2 h/day)**									
≥2 h/day	260 (47.3)	191 (34.5)	<0.001	195 (47.2)	219 (64.4)	<0.001	186 (45.5)	195 (55.4)	0.006
**Parent-reported SHS exposure**									
Any (≥1 time/week)	81 (14.7)	24 (4.3)	<0.001	57 (13.8)	25 (7.4)	0.005	71 (17.4)	48 (13.6)	0.159
**Urinary cotinine**									
≥LOD	435 (79.1)	534 (96.4)	<0.001	320 (77.5)	320 (94.1)	<0.001	342 (83.6)	331 (94.0)	<0.001
≥3 ng/mL	78 (14.2)	167 (30.1)	<0.001	79 (19.1)	104 (30.6)	<0.001	86 (21.0)	119 (33.8)	<0.001

Variables	3-5 years			6-8 years			9-11 years		
	Cycle 3 (N=550)	Cycle 4 (N=554)	p	Cycle 3 (N=413)	Cycle 4 (N=340)	p	Cycle 3 (N=409)	Cycle 4 (N=352)	p
	n (%)	n (%)		n (%)	n (%)		n (%)	n (%)	
≥5 ng/mL	21 (3.8)	49 (8.8)	<0.001	24 (5.8)	37 (10.9)	0.011	21 (5.1)	47 (13.4)	<0.001
Geometric mean (95% CI)	0.97 (0.91, 1.04)	1.82 (1.70, 1.95)	<0.001	1.09 (1.00, 1.20)	1.50 (1.35, 1.65)	<0.001	1.25 (1.15, 1.36)	1.67 (1.53, 1.83)	<0.001

Categorical variables are presented as n (%). Paternal and maternal smoking categories were based on current smoking at the time of the survey; non-smokers included never and former smokers. Smoke-free denotes no current parental smoker; smoking denotes at least one current parental smoker. Cigarettes smoked per day among paternal smokers are presented as mean (standard error [SE]) and were compared between cycles using independent-samples t tests within each age group. Urinary cotinine concentrations are presented as creatinine-adjusted geometric means (GMs) with 95% confidence intervals (CIs), estimated from generalized linear models of log-transformed urinary cotinine with log-transformed urinary creatinine included as a covariate. Categorical variables were compared using Pearson's chi-squared tests, and the p-value for the GM row was obtained from the survey-cycle term in the corresponding model within each age group. Cycle 3 was conducted in 2016-2017 and Cycle 4 in 2019-2020. Within Cycle 4, children aged 6-8 and 9-11 years were surveyed in 2019, whereas children aged 3-5 years were surveyed in 2020. Analyses used unweighted data and are not nationally representative. N=2618 because one participant had missing time-activity data; this participant was retained in the primary analyses (N=2619).
CI: confidence interval; GM: geometric mean; KoNEHS: Korean National Environmental Health Survey; LOD: limit of detection; SE: standard error; SHS: secondhand smoke.

Supplementary Table S4. Exploratory interaction models of survey cycle with age group and paternal smoking intensity among children aged 3-11 years in a cross-sectional secondary analysis of KoNEHS Cycles 3 and 4, South Korea, 2016-2017 and 2019-2020 (N=2619)

**Variable**	**β (95% CI)**	**p**
**A. Age group**		
3-5 years vs. 9-11 years	0.14 (0.03, 0.25)	0.014
6-8 years vs. 9-11 years	0.05 (-0.06, 0.17)	0.371
Survey cycle × 3-5 years	0.36 (0.20, 0.52)	<0.001
Survey cycle × 6-8 years	0.02 (-0.15, 0.20)	0.783
Overall interaction		<0.001
**B. Paternal smoking intensity**		
Moderate smoker vs. Non-smoker	0.14 (0.04, 0.24)	0.008
Heavy smoker vs. Non-smoker	0.35 (0.21, 0.48)	<0.001
Survey cycle × Moderate smoker	0.01 (-0.13, 0.16)	0.846
Survey cycle × Heavy smoker	-0.27 (-0.48, -0.05)	0.016
Overall interaction		0.039

Linear regression models used log-transformed urinary cotinine as the outcome and included interaction terms between survey cycle and (A) age group or (B) paternal smoking intensity. β coefficients are presented on the log-cotinine scale. Both models included survey cycle, age group, sex, housing type, building age, household income, education and occupation of both parents, paternal smoking, maternal smoking, vigorous physical activity, current allergic disease, home ventilation frequency, and log-transformed urinary creatinine, together with the relevant interaction terms. Cycle 3, children aged 9-11 years, and paternal non-smokers were the reference categories. Smoking categories were based on current smoking status at the time of the survey; non-smokers included never and former smokers. Main-effect coefficients for age group and paternal smoking intensity therefore represent the corresponding contrasts in Cycle 3, whereas interaction coefficients represent the additional change in those contrasts in Cycle 4. Overall interaction p-values were obtained from Type 3 tests of the joint interaction terms. Given multiple comparisons and the absence of prespecified directional hypotheses, these analyses were exploratory, and individual p-values were treated as nominal and interpreted descriptively. Analyses used unweighted pooled data from KoNEHS Cycles 3 (2016-2017) and 4 (2019-2020) and are not nationally representative.
CI: confidence interval; KoNEHS: Korean National Environmental Health Survey; β: regression coefficient.

Supplementary Table S5. Cycle-specific weighted and unweighted sensitivity analyses of log-transformed urinary cotinine among children aged 3-11 years in a cross-sectional secondary analysis of KoNEHS Cycles 3 and 4, South Korea (Cycle 3: 2016-2017, N=1372; Cycle 4: 2019-2020, N=1246)

Variable	Cycle 3 (N=1372)				Cycle 4 (N=1246)			
	Unweighted		Weighted		Unweighted		Weighted	
	β (95% CI)	p	β (95% CI)	p	β (95% CI)	p	β (95% CI)	p
**Age group**								
3-5 years (vs. 9-11 years)	0.15 (0.01, 0.29)	0.031	0.20 (0.03, 0.37)	0.022	0.45 (0.32, 0.59)	<0.001	0.45 (0.29, 0.61)	<0.001
6-8 years (vs. 9-11 years)	0.04 (-0.09, 0.17)	0.555	0.07 (-0.05, 0.19)	0.268	0.08 (-0.04, 0.20)	0.188	0.06 (-0.07, 0.20)	0.368
**Paternal smoking**								
Moderate smoker (vs. Non-smoker)	0.12 (0.01, 0.23)	0.030	0.13 (0.02, 0.25)	0.026	0.15 (0.05, 0.25)	0.002	0.10 (-0.02, 0.22)	0.100
Heavy smoker (vs. Non-smoker)	0.35 (0.20, 0.50)	<0.001	0.35 (0.21, 0.50)	<0.001	0.09 (-0.06, 0.25)	0.249	0.05 (-0.12, 0.23)	0.535
**Weekday indoor time at home**								
≥15 h/day (vs. <15 h/day)	0.01 (-0.11, 0.12)	0.905	0.00 (-0.13, 0.12)	0.965	0.04 (-0.06, 0.15)	0.416	0.07 (-0.08, 0.21)	0.368
**Weekday kindergarten/school time**								
≥7 h/day (vs. <7 h/day)	-0.17 (-0.28, -0.05)	0.004	-0.19 (-0.32, -0.05)	0.007	-0.07 (-0.17, 0.04)	0.225	-0.04 (-0.16, 0.09)	0.588
**Weekday indoor time at other locations**								
≥2 h/day (vs. <2 h/day)	-0.07 (-0.20, 0.05)	0.230	-0.07 (-0.22, 0.07)	0.309	-0.02 (-0.14, 0.10)	0.740	-0.02 (-0.16, 0.12)	0.786
**Weekend indoor time at home**								
≥19 h/day (vs. <19 h/day)	0.00 (-0.11, 0.11)	0.946	0.01 (-0.15, 0.16)	0.943	0.02 (-0.08, 0.12)	0.732	0.02 (-0.10, 0.14)	0.732
**Weekend indoor time at other locations**								
≥2 h/day (vs. <2 h/day)	0.03 (-0.07, 0.14)	0.548	0.06 (-0.07, 0.19)	0.343	-0.13 (-0.23, -0.03)	0.010	-0.15 (-0.27, -0.03)	0.018

Variable	Cycle 3 (N=1372)	Cycle 4 (N=1246)

Analyses were conducted separately for Cycle 3 (2016-2017; N=1372) and Cycle 4 (2019-2020; N=1246). Weighted models incorporated the cycle-specific survey weights provided by KoNEHS and accounted for the corresponding strata and primary sampling clusters, whereas unweighted models did not incorporate these survey-design features. Weighted and unweighted estimates are presented side by side to evaluate sensitivity to survey weighting. Selected coefficients are shown. Values are β coefficients with 95% confidence intervals (CIs) from linear regression models of log-transformed urinary cotinine. Exponentiating β yields the corresponding geometric mean ratio (GMR). Models were adjusted for age group, sex, weekday indoor time at home, weekday kindergarten/school time, weekday indoor time at other locations, weekend indoor time at home, weekend indoor time at other locations, vigorous physical activity, housing type, building age, household income, education and occupation of both parents, paternal and maternal smoking, home ventilation frequency, current allergic disease, and log-transformed urinary creatinine. Survey cycle was not included because models were fitted separately for each cycle. Smoking categories were based on current smoking status at the time of the survey; non-smokers included never and former smokers. The sensitivity analyses included 2618 participants because one participant with missing time-activity data was excluded; this participant was retained in the primary analyses (N=2619). Because cycle-specific sampling weights are not harmonizable across cycles, the pooled main analyses used unweighted data.

CI: confidence interval; GMR: geometric mean ratio; KoNEHS: Korean National Environmental Health Survey; β: regression coefficient.
